# Historical Trends in PM_2.5_-Related Premature Mortality during 1990–2010 across the Northern Hemisphere

**DOI:** 10.1289/EHP298

**Published:** 2016-08-19

**Authors:** Jiandong Wang, Jia Xing, Rohit Mathur, Jonathan E. Pleim, Shuxiao Wang, Christian Hogrefe, Chuen-Meei Gan, David C. Wong, Jiming Hao

**Affiliations:** 1 U.S. Environmental Protection Agency, Research Triangle Park, North Carolina, USA; 2 State Key Joint Laboratory of Environmental Simulation and Pollution Control, School of Environment, Tsinghua University, Beijing, China

## Abstract

**Background:**

Air quality across the northern hemisphere over the past two decades has witnessed dramatic changes, with continuous improvement in developed countries in North America and Europe, but a contrasting sharp deterioration in developing regions of Asia.

**Objective:**

This study investigates the historical trend in the long-term exposure to PM_2.5_ and PM_2.5_-related premature mortality (PM_2.5_-mortality) and its response to changes in emission that occurred during 1990–2010 across the northern hemisphere. Implications for future trends in human exposure to air pollution in both developed and developing regions of the world are discussed.

**Methods:**

We employed the integrated exposure–response model developed by Health Effects Institute to estimate the PM_2.5_-mortality. The 1990–2010 annual average PM_2.5_ concentrations were obtained from the simulations using the WRF-CMAQ model. Emission mitigation efficiencies of sulfur dioxide (SO_2_), nitrogen oxides (NO_x_), ammonia (NH_3_), and primary PM are estimated from the PM_2.5_-mortality responses to the emission variations.

**Results:**

Estimated PM_2.5_-mortalities in East Asia and South Asia increased by 21% and 85% respectively, from 866,000 and 578,000 in 1990, to 1,048,000 and 1,068,000 in 2010. PM_2.5_-mortalities in developed regions (i.e., Europe and high-income North America) decreased substantially by 67% and 58% respectively.

**Conclusions:**

Over the past two decades, correlations between population and PM_2.5_ have become weaker in Europe and North America due to air pollution controls but stronger in East Asia due to deteriorating air quality. Mitigation of primary PM appears to be the most efficient way for increasing health benefits (i.e., providing the largest mortality reduction per unit emissions). However, reductions in emissions of NH_3_ are needed to maximize the effectiveness of NO_x_ emission controls.

**Citation:**

Wang J, Xing J, Mathur R, Pleim JE, Wang S, Hogrefe C, Gan CM, Wong DC, Hao J. 2017. Historical trends in PM_2.5_-related premature mortality during 1990–2010 across the northern hemisphere. Environ Health Perspect 125:400–408; http://dx.doi.org/10.1289/EHP298

## Introduction

Fine particles—those defined as aerosols with aerodynamic diameter ≤ 2.5 μm (PM_2.5_)—in the atmosphere have been well known for their potential negative impacts on human health that can affect the respiratory, cardiovascular, and cerebrovascular systems. The robust association between ambient PM_2.5_ mass concentration and public health has been demonstrated by epidemiologic studies across the world (Pope et al. 1995; Katsouyanni et al. 2001; Pope and Dockery 2006; Chen et al. 2011), but most previous studies focused on a limited range of ambient annual average concentrations, typically from approximately 5 μg/m^3^ to 30 μg/m^3^, in developed countries (Dockery et al. 1993; Pope et al. 1995; Pope and Dockery 2006). Recent studies (e.g., Pope et al. 2009, 2011; Burnett et al. 2014) enabled the extension of the traditional calculation method for global scale applications by using increments of cigarette smoking as equivalent concentration of PM_2.5_. As one example, the integrated exposure–response model (IEM) reported in the Global Burden of Disease study (Burnett et al. 2014) was successfully applied in an estimation of health impacts of long-term exposures to PM_2.5_ at the global scale: The result suggests the number of deaths attributable to ambient PM_2.5_ was 3.2 million worldwide in 2010 (Lim et al. 2012).

Assessment of the avoided human health impacts associated with air quality improvements is critical for the design and implementation of air pollution control policy. Some studies have estimated air quality changes and health benefits from emission mitigations by using sensitivity analysis with a chemical transport model. For example, Fann et al. (2009) quantified the health benefits from controls on different locations, sources, and emission types by employing the Community Multiscale Air Quality (CMAQ) response surface model. They further characterized the PM_2.5_ air quality impacts and human health benefits from 17 emission sectors across the contiguous United States by using the Comprehensive Air Quality Model with Extensions (CAMx) source apportionment air quality modeling techniques (Fann et al. 2012). Lee et al. (2015) calculated the sensitivities of global PM_2.5_-related premature mortality (referred to as PM_2.5_-mortality in this article) to emissions by using the adjoint of the GEOS-Chem chemical transport model (Lee et al. 2015; Henze et al. 2007). Lelieveld et al. (2015) used the zero-out method in a global atmospheric chemistry model to investigate the link between premature mortality and seven emission source categories for conditions representative of 2010. To date, however, little effort has been devoted toward assessing long-term exposure to PM_2.5_ and health impacts associated with recent historical trends in air pollution. Compared to the sensitivity analysis that relies heavily on the baseline situation (i.e., the nonlinearly responding functions), a historical trend analysis is better constrained, since it is based on actual documented changes. However, a challenge with the approach is the quantification of a reliable historical trend in PM_2.5_ concentrations with adequate spatial resolution.

Anthropogenic emissions of primary aerosol and gaseous precursors of PM_2.5_ have witnessed dramatic changes over the past two decades across the northern hemisphere. During the period 1990–2010, sulfur dioxide (SO_2_) and nitrogen oxides (NO_x_) emissions across the United States decreased by about 66% and 50%, respectively (e.g., Xing et al. 2013), while emissions have increased dramatically in many developing regions. These changing emissions have resulted in contrasting trends in the regional aerosol burden across the northern hemisphere. Understanding the historical trend in long-term exposure to PM_2.5_, as well as the health benefits from the air pollution controls in the developed countries and the health risks arising from the deterioration of air quality in developing countries, is crucial for policy design in the future.

This study aims to provide an estimation of historical trends in long-term exposure to PM_2.5_ and its impact on PM_2.5_-mortality during 1990–2010 across the northern hemisphere. We first detail the method used to calculate PM_2.5_-mortality and emission mitigation efficiency (EME). The estimated long-term exposure to PM_2.5_ and mortality and their response to changes in emissions is then quantified. Uncertainties and implications for future trends in air pollution exposure are also discussed.

## Methods

### Historical Emission and PM_2.5_ Concentration Trend

Historical emission trends of gaseous pollutants and primary particles used in this study are derived from the combination of the year-specific inventory from the Emissions Database for Global Atmospheric Research (version 4.2; EDGAR) (EC-JRC 2011) for the period 1990–2008 and an extrapolation for the years of 2009 and 2010 based on recent estimates of activity change (Xing et al. 2015b). The SO_2_, NO_x_, volatile organic compounds (VOC), and primary PM emissions in Europe and high-income North America (includes Canada and the United States) (Naghavi et al. 2015) have seen continuous reductions by 5.4/5.4%, 1.5/1.8%, 3.3/3.3%, and 4.8/4.6% per year, respectively, during 1990–2010. In contrast, SO_2_, NO_x_, and VOC emissions in East Asia have increased continuously by 3.2%, 4.2%, and 2.3% per year, respectively. Stricter controls on primary PM emissions have been implemented in China since 2003 (Wang and Hao 2012), thus its positive (+) 0.3% per year increase rate is much smaller compared to gaseous pollutants. Ammonia (NH_3_) emissions exhibit an increasing trend both in China (+2.6% per year increase) and in the United States (+1.6% per year increase) but exhibit a declining (–) trend in Europe (–1.0% per year) due to stricter controls. Additional details on the emission processing for the model simulations are provided in Xing et al. (2015b).

**Figure 1:**
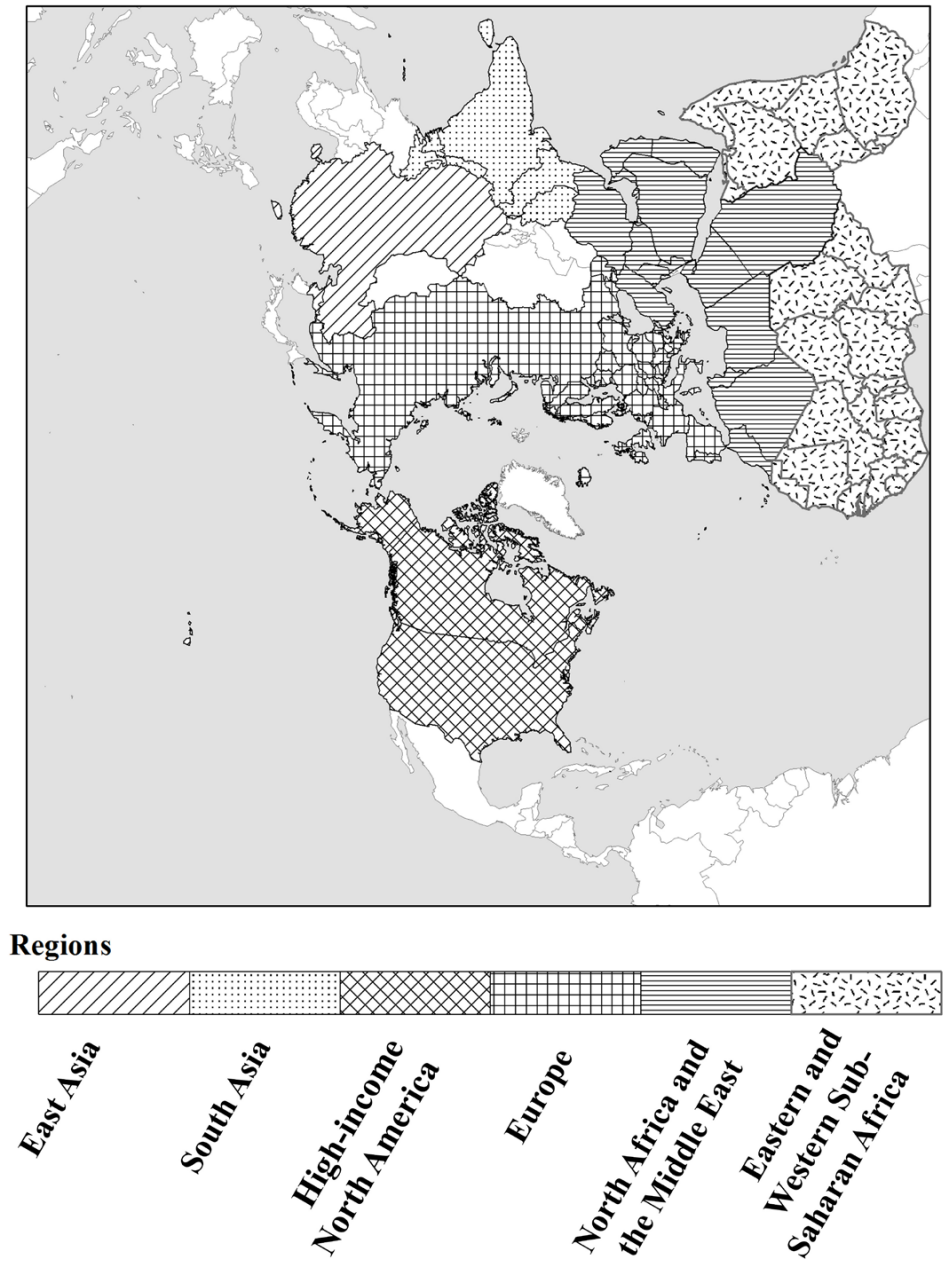
The northern hemisphere model simulation domain and the six selected subregions used in the analysis: East Asia, high-income North America, Europe, South Asia, North Africa and the Middle East, eastern and western sub-Saharan Africa. The map used in this figure was created using ArcGIS software by Esri (http://www.esri.com).

The 1990–2010 gridded annual average PM_2.5_ concentrations with 108 km × 108 km resolution over the northern hemisphere (see domain coverage presented in Figure 1) are obtained from simulations with a hemispheric version of the Weather Research and Forecast (WRF) model coupled with the CMAQ model developed by the U.S. Environmental Protection Agency (EPA) (Wong et al. 2012; Wang et al. 2014). CMAQ is a sophisticated modeling system which has multiple capabilities of simulating concentrations of fine particulate and other air pollutants in the atmosphere involving complex pollutant interactions on urban (e.g., Xing et al. 2011), regional (e.g., Appel et al. 2008) and hemispheric (Mathur et al. 2012, 2014) scales. An extensive examination of the model’s ability to capture trends in gaseous precursors, PM_2.5_ chemical composition and aerosol burden has been conducted through comparison with multi-decadal trends in observations. First, the ability of WRF-CMAQ in reproducing the historical trend in AOD over the northern hemisphere has been quantitatively evaluated through a comparison of simulated values over the 21-years with six satellite-retrieved AOD products including AVHRR, TOMS, SeaWiFS, MISR, MODIS-Terra and MODIS-Aqua as well as long-term historical records from 11 AERONET sites during the 1990–2010 period (Xing et al. 2015a). Second, the model performance for simulation of gaseous species and PM_2.5_ composition was future evaluated through comparison with measurements at several ground observation networks mostly over Europe and North America over the past two decades. Results suggested that model simulated ambient PM_2.5_ trends over the past two decades largely agree with those derived from observations (Xing et al. 2015b).

### Estimates of Health Impacts Due to Long-Term Exposure to PM_2.5_

The PM_2.5_-mortality estimated in this study includes five causes of premature mortality: ischemic heart disease (IHD), cerebrovascular disease (stroke), chronic obstructive pulmonary disease (COPD), lung cancer (LC) for adults > 25 years old, and acute respiratory lung infection (ALRI) for children < 5 years old. The method is based on the algorithm in the Environmental Benefits Mapping and Analysis Program-Community Edition (BenMAP CE) (e.g., Wang et al. 2015), released by the U.S. EPA, and the Integrated exposure–response model from global burden of disease (GBD) (Lim et al. 2012; Burnett et al. 2014).

The concentration–response function (Equation 1) is summarized as below:

*Mortality_PM_*_2.5_ = Σ*_i_*_=_ *_IHD, stroke, COPD, LC, ALRI_ incidence*_0,_ *_i_* × *PAF_i_* × *Population* [1]

in which, *incidence*_0_ *_,i_* is the baseline incidence rate of the cause-specific premature mortality of *i*. The value of *incidence*_0_ *_,i_* is based on Naghavi et al. (2015). *PAF_i_* is population attributable fraction (PAF) of the cause-specific premature mortality of *i*. The value of *PAF* is defined by Equation 2:

*PAF_i_* = (*RR_i_ – 1*)/*RR_i_* [2]

where *RR_i_* is the relative risk for the cause-specific premature mortality of *i* (Equation 3):


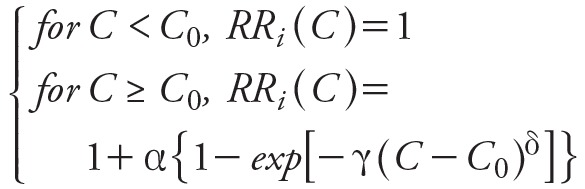
_[3]_

where, *C* is the ambient PM_2.5_ concentration, and *C*_0_ is the threshold value of PM_2.5_ concentration below which there is no additional risk assumed in this study. 〈, γ, and δ are parameters that determine the overall shape of the concentration–response relationship (Burnett et al. 2014). In the calculations presented here, the estimates of relative risks (i.e., *RR_i_* in Equation 2) were obtained from the look-up table developed by Apte et al. (2015), which reports RR for PM_2.5_ concentrations in the 0-410 μg/m^3^ range in 0.1 μg/m^3^ steps.

The gridded population data are interpolated from the 1990, 1995, 2000, 2005, and 2010 Gridded Population of the World (version 3; GPW) (CIESIN and CIAT 2005). The population age structures were obtained from Ahmad et al. (2001). The original population data on a 0.25° × 0.25° grid was apportioned to individual grid pixels matching the model grid (108 km × 108 km). The population-weighted average PM_2.5_ is estimated from the annual average PM_2.5_ concentrations weighted by the population. To quantify the correlations between population and PM_2.5_, we defined the ratio of population-weighted average PM_2.5_ to regional average PM_2.5_ as the population scale factor (PSF). Thus, a large PSF (> 1) indicates a positive correlation between population and PM_2.5_, while a small PSF (< 1) indicates a negative correlation between population and PM_2.5_.

### Calculation of EME

EME, an index to represent the health benefits from unit emission reduction in different source categories, has previously been estimated using sensitivity analysis methods (Fann et al. 2009, 2012; Lee et al. 2015; Lelieveld et al. 2015). In this study, we utilized the changes in air pollution-attributed health effects and emissions over the past two decades to estimate the EME. The simulation of year 1990 is defined as the baseline scenario. Each of the other years simulated from 1991 to 2010 then represents 20 scenarios with different emission and meteorological conditions relative to 1990. A principle similar to that in source apportionment methods was adopted; changes in concentrations of inorganic aerosols (i.e., sulfate, nitrate, and ammonium) are attributed to changes in emissions of their corresponding precursors (i.e., SO_2_, NO_x_, and NH_3_, respectively), though it is important to note that this is only a first-order approximation due to the nonlinear coupling of the atmospheric SO_x_–NO_x_–NH_x_ system. Changes in concentrations of other inorganic particles (excluding sulfate, nitrate, and ammonium) as well as primary organic aerosols are attributed to the change in their emissions (noted as primary PM). Since PM_2.5_ mass is still considered the most robust indicator of mortality impacts in epidemiologic cohort studies of long-term exposure (Chen et al. 2008), the assumption here is that the health effects of exposure to PM_2.5_ are independent of source and composition (Lee et al. 2015). Thus, the impact of various pollutants on mortality are estimated to be proportional to their contribution to PM mass. However, considering the non-linearity of the risk-curve, these estimates could be considered to be on the higher side for cases where one component changes but the others are constant. The year specific EME for each year in the period 1991–2010 relative to the base year of 1990, for an aerosol species, *p* (where *p* = SO_2_, NO_x_, NO_3_, or primary PM), is then estimated as follow:


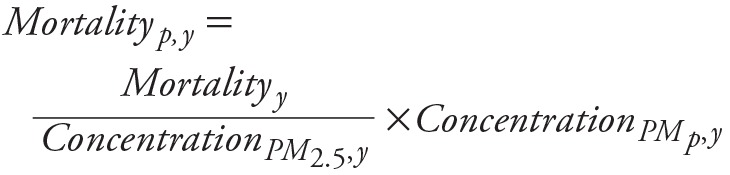
_[4]_

where *y* = 1990…2010 and PM*_p_* represents SO_4_^2–^, NO_3_^–^, NH_4_^+^, or other inorganic particles and primary organic aerosols, and


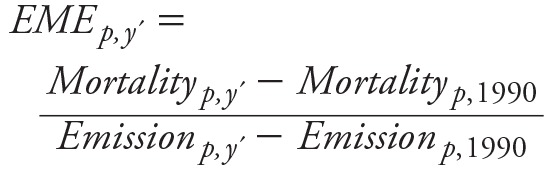
_[5]_

where *y*´ = 1991…2010.

To limit the influence of year-to-year variations in meteorological condition, the 21-year average EME for a particular species was estimated from a linear regression of the changes in PM_2.5_-mortality and emissions of 1991–2010 relative to 1990.

## Results

### Population-Weighted PM_2.5_ and Population Scaled Factor (PSF)

**Figure 2:**
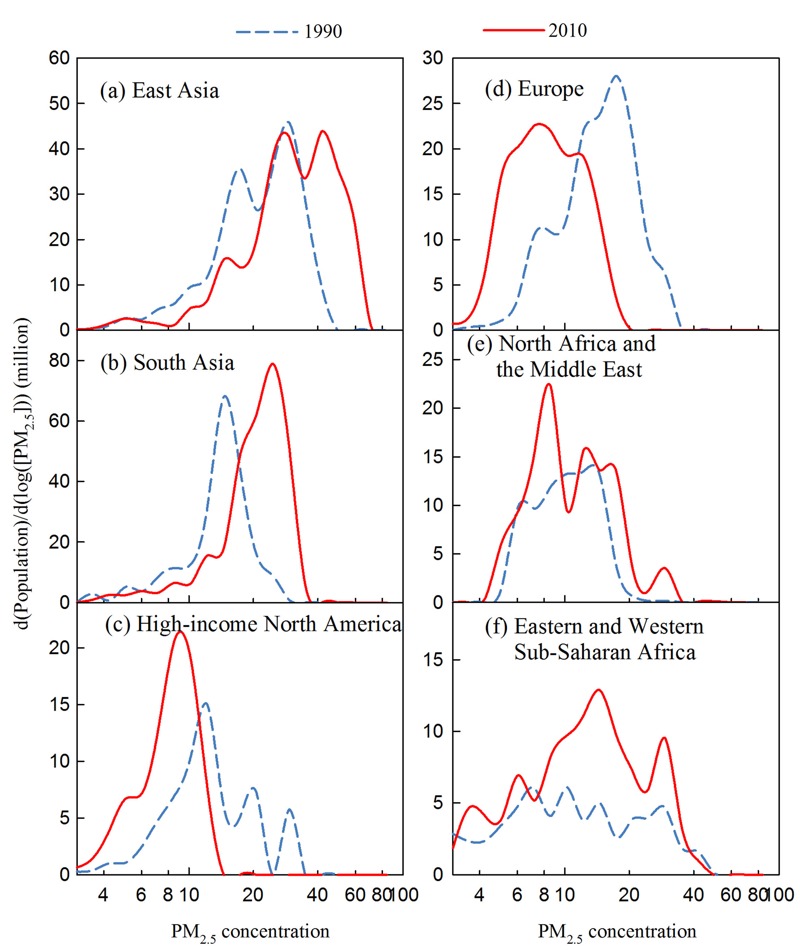
Changing population exposure to ambient PM_2.5_ levels across the six subregions used in the analysis: (*a*) East Asia, (*b*) South Asia, (*c*) high-income North America, (*d*) Europe, (*e*) North Africa and Middle East, and (*f*) eastern and western sub-Saharan Africa, of the northern hemisphere (shown in Figure 1), for the years 1990 (blue dash line) and 2010 (red solid line): Note: Model estimated surface PM_2.5_ concentration across grid cells in a region are grouped by population distributions during 1990–2010. d[Population]/d[log(PM_2.5_)] represents the population per unit PM_2.5_ section in log scale. The area below a curve represents the total population for that region for that year.

We focus our analysis on six selected regions within the northern hemisphere simulation domain displayed in Figure 1, including two highly populated and polluted regions (East Asia and South Asia), two well-developed regions (Europe and high-income North America), and two regions within or downwind of deserts (North Africa and the Middle East, eastern and western sub-Saharan Africa). The distribution of surface PM_2.5_ concentrations across grid cells grouped by population in six regions is presented in Figure 2. In East Asia and South Asia, most of the population is concentrated in the areas with high annual average PM_2.5_ concentrations exceeding 10 μg/m^3^, which is the World Health Organization’s air quality guideline for PM_2.5_ (WHO 2016). Additionally, compared to 1990, in 2010 a larger population (i.e., area under the curve) was exposed to higher PM_2.5_ levels, suggesting that greater health risks due to PM_2.5_ likely occurred in recent years in these two regions. In the well-developed regions (i.e., Europe and high-income North America), population exposure to high PM_2.5_ levels (> 10 μg/m^3^) was much greater in 1990 than in 2010, indicating the health benefits from air quality improvement resulting from emission reductions. In the two desert regions (i.e., North Africa and the Middle East, eastern and western sub-Saharan Africa), the range of PM_2.5_ levels to which populations are exposed were similar in 1990 and 2010, but due to population growth, a much greater number of people were exposed to PM_2.5_ in 2010 than in 1990. Also, the increased populations were mainly concentrated in the areas with high PM_2.5_ levels (> 10 μg/m^3^), suggesting greater health risks due to PM_2.5_.

**Figure 3:**
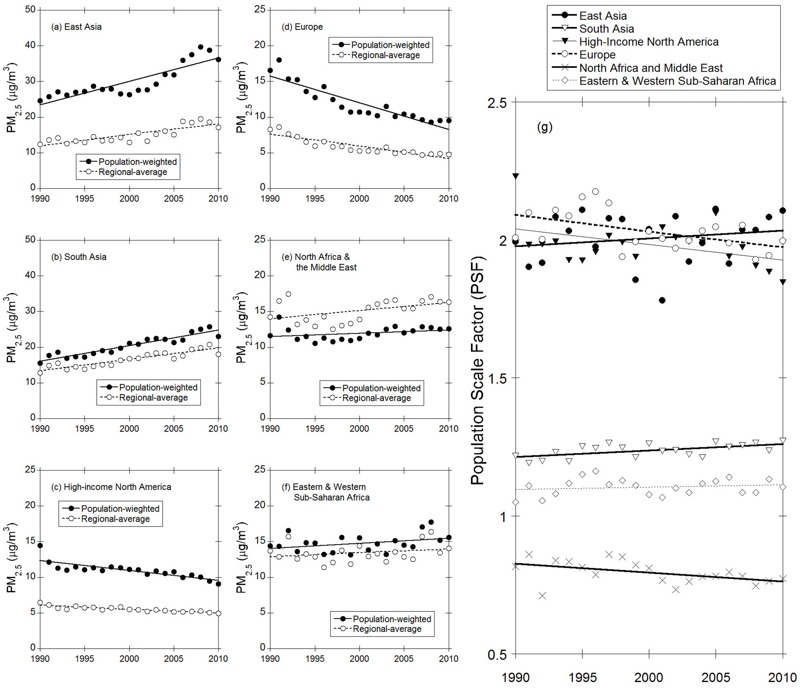
Comparison of long-term trends (1990–2010) of population-weighted and regional average PM_2.5_ concentrations for the six regions: (*a*) East Asia, (*b*) South Asia, (*c*) high-income North America, (*d*) Europe, (*e*) North Africa and the Middle East, (*f*) eastern and western sub-Saharan Africa, and (*g*) the population scale factor (PSF). Units for both the population-weighted and regional average PM_2.5_ concentrations are shown in μg/m^3^. The population scale factor is the ratio of the population-weighted average PM_2.5_ to the regional average PM_2.5_.

To further investigate the correlation between PM_2.5_ and population in six typical regions, Figure 3 presents comparisons of the historical trend of population-weighted and regional average PM_2.5_ concentrations from 1990 to 2010. Not surprisingly, contrasting trends in PM_2.5_ concentrations were found between developing and developed regions. These trends are comparable with trends in exposure estimates recently suggested in van Donkelaar et al. (2015). Developing regions of East Asia and South Asia exhibit continually increasing trends in regional average PM_2.5_ concentrations of +0.66 and +0.43 μg/m^3^ per year, respectively, while developed regions, including Europe and high-income North America, have experienced decreasing trends in regional average PM_2.5_ concentrations of –0.37 and –0.14 μg/m^3^ per year, respectively. The population-weighted PM_2.5_ in East Asia and South Asia increased from 24.6 and 15.6 μg/m^3^ in 1990 to 36.1 and 23.0 μg/m^3^ in 2010, while the population-weighted PM_2.5_ in Europe and high-income North America decreased from 16.6 and 14.5 μg/m^3^ in 1990 to 9.6 and 9.1 μg/m^3^ in 2010.

Additionally, for all regions except North Africa and the Middle East, the population-weighted PM_2.5_ concentrations are always higher than the regional averages. This can be explained by the positive correlation in spatial distributions between population and PM_2.5_ concentrations. In regions where ambient PM_2.5_ is mainly caused by anthropogenic sources, populated areas are usually also polluted, leading to a positive correlation between population and PM_2.5_. However, in regions where natural sources (wind-blown dust) are dominant (e.g., North Africa and the Middle East), since dust areas are not amenable for living, a negative spatial correlation between population and PM_2.5_ concentrations is expected, resulting in lower population-weighted PM_2.5_ compared to the regional averages.

The PSF value can be used to measure the extent of correlation between population and PM_2.5_. Large PSF (> 1) indicate a positive correlation between population and PM_2.5_. Figure 3g summarizes the PSF trends in the six regions. Large 21-year average PSF (around 2.0) are found in East Asia, Europe and high-income North America. Small PSF are found in North Africa and the Middle East (around 0.79), indicating a negative correlation between the population and PM_2.5_ concentration. Additionally, contrasting PSF trends during 1990–2010 are evident in the six regions. The PSF trends are statistically significant at the 95% confidence level (*p* = 0.05) in all regions except East Asia and eastern and western sub-Saharan Africa. In East Asia and South Asia, the simultaneous growth of population and PM_2.5_ concentrations during the past two decades has led to an increasing trend in PSF from 1.99 and 1.22, respectively, in 1990 to 2.11 and 1.27, respectively, in 2010, suggesting more serious health impacts from exposure to air pollution. Conversely, Europe and high-income North America exhibit decreasing trends in PSF, suggesting increasing health benefits from air pollution controls.

### Estimated PM_2.5_-Mortality

The estimated annual average PM_2.5_-mortality for 1990 and 2010 and its change from 1990 to 2010 in the six regions are summarized in Table 1. Among the six regions, estimated PM_2.5_-mortality in 2010 was highest in two populated regions with poor air quality, (i.e., East and South Asia), where the PM_2.5_-mortality increased by 21% and 85%, respectively, from 866,000 and 578,000 in 1990, to 1,048,000 and 1,068,000 in 2010. North Africa and the Middle East and eastern and western sub-Saharan Africa also experienced a substantial growth of mortality risk due to PM_2.5_ over the past two decades, with increases in PM_2.5_-mortality of 38% and 53%, respectively. However, in developed regions, including high-income North America and Europe, the PM_2.5_-mortality decreased by 58% and 67%, respectively, in 2010 compared to 1990. The estimates of PM_2.5_-mortality in this study are comparable in magnitude with results in a previous study (Lim et al. 2012) as shown in Table 1. The differences in estimates compared to Lim et al. (2012) arise primarily due to differences in the spatial resolution employed in this analysis, since the non-linearity of the risk function enlarges the uncertainties of PM_2.5_-mortality estimates related to the exposure allocation.

**Table 1:** Estimated PM_2.5_-mortality due to long-term exposure to PM_2.5_ in 1990 and 2010 (in thousands).

Year	Case	East Asia	South Asia	High-income North America	Europe	North Africa and the Middle East	Eastern and western sub-Saharan Africa
1990	Lim et al. (2012)^*a*^	947	538	163	729	136	97
	This study^*b*^	866	578	122	418	124	96
2010	Lim et al. (2012)	1,271	771	110	420	176	105
	This study	1,048	1,068	51	137	171	147
Incr = (Y_2010_/Y_1990_ – 1)	Lim et al. (2012)	34%	43%	–33%	–42%	29%	8%
	This study	21%	85%	–58%	–67%	38%	53%
^***a***^Data obtained from IHME (2015). ^***b***^Estimated PM_2.5_-mortality in this study.							

The PM_2.5_-mortality is estimated from the product of three factors (see Equation 1): population, PAF (which depends on PM_2.5_ concentrations), and baseline incidence rate of the cause-specific mortality (influenced by living conditions, access to medical care). Thus, the trend in PM_2.5_-mortality can be driven by changes in these three factors. To estimate the relative contributions of these three factors to changes in PM_2.5_-mortality, we designed three control scenarios. Impacts due to the change of PAF were estimated from the difference between the base case and a control case in which population and baseline incidence rate of the cause-specific mortality were the same as the base case, but the 2010 PM_2.5_ concentration was set to the 1990 PM_2.5_ concentration.

Similarly, impacts due to change in population (or due to change of baseline incidence rate of the cause-specific mortality) were estimated from the difference between the base case and the control case in which the population (or baseline incidence rate of the cause-specific mortality) in 2010 was set to that of 1990, but the other two factors were kept the same as in the base case.

**Figure 4:**
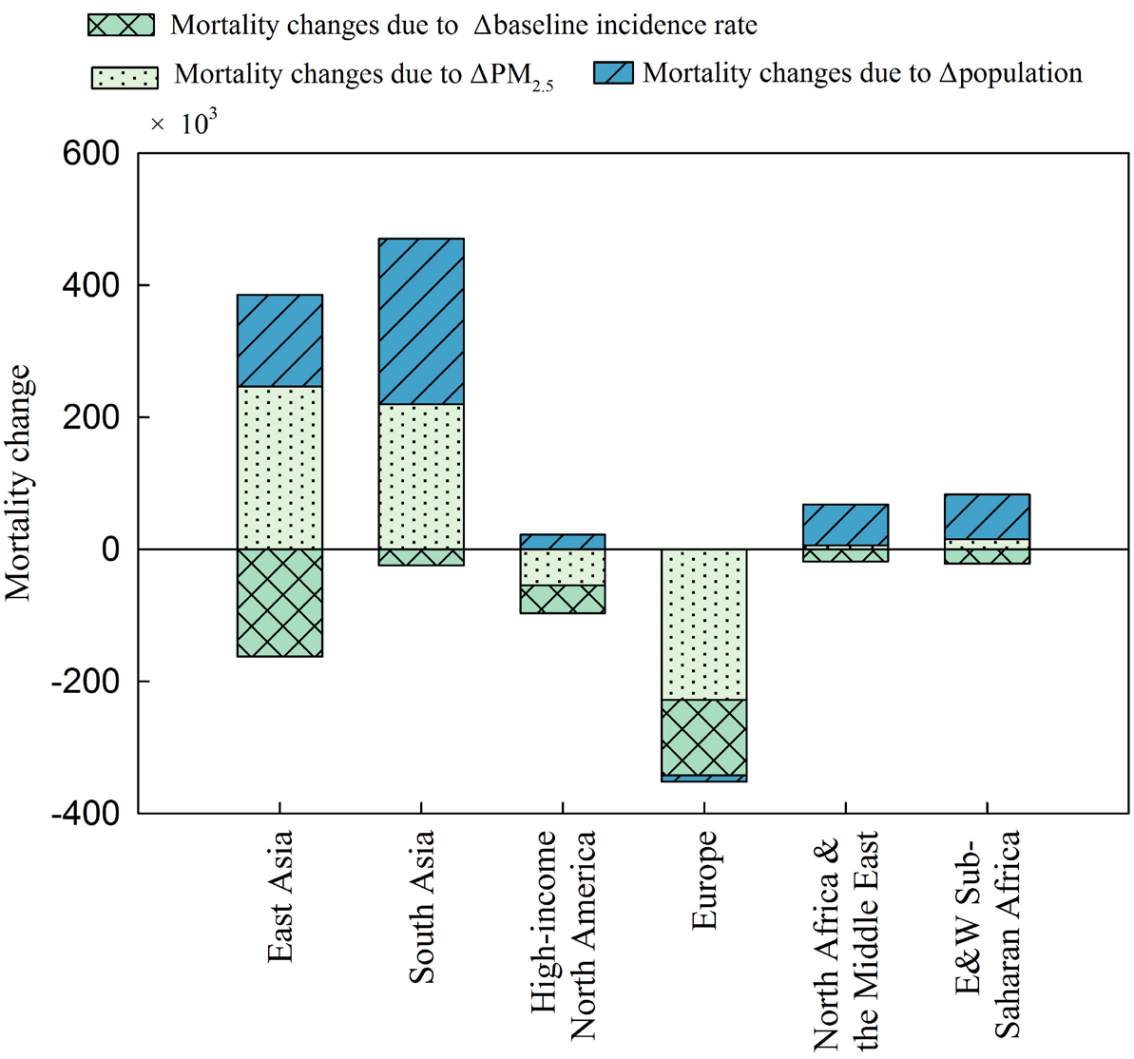
Relative contributions of the baseline incidence rate, changes in population, and changes in PM_2.5_ concentrations to the estimated PM_2.5_-mortality changes during 1990–2010 in the different regions across the northern hemisphere. Note: Positive numbers indicate an increase in estimated mortality due to exposure to ambient PM_2.5_, while negative numbers represent a reduction.

Figure 4 displays the changes in PM_2.5_-mortality due to three factors in six regions. In East Asia and South Asia, the increase in PM_2.5_ concentration caused an increase in mortalities of 247,000 and 219,000, respectively. The benefits for the PM_2.5_-mortality reduction from the improvement in living conditions and the quality of medical care has been offset by the increased health risk from the deterioration of air quality. Compared to East Asia, the total increase of PM_2.5_-mortality in South Asia is larger because of a larger contribution from population growth and a smaller reduction from the improvement of living conditions and the quality of medical care. In Europe and high-income North America, the decrease in PM_2.5_ concentrations is the major reason for the reduction in PM_2.5_-mortality, overwhelming the impact from the other two factors. In the two desert areas (i.e., North Africa and the Middle East and eastern and western sub-Saharan Africa), changes in PM_2.5_-mortality are dominated by population growth. A small fraction of the growth of PM_2.5_-mortality during 1990–2010 was caused by the deterioration in local air quality.

### Response of PM_2.5_-Mortality to Changes in Local Emissions

**Figure 5:**
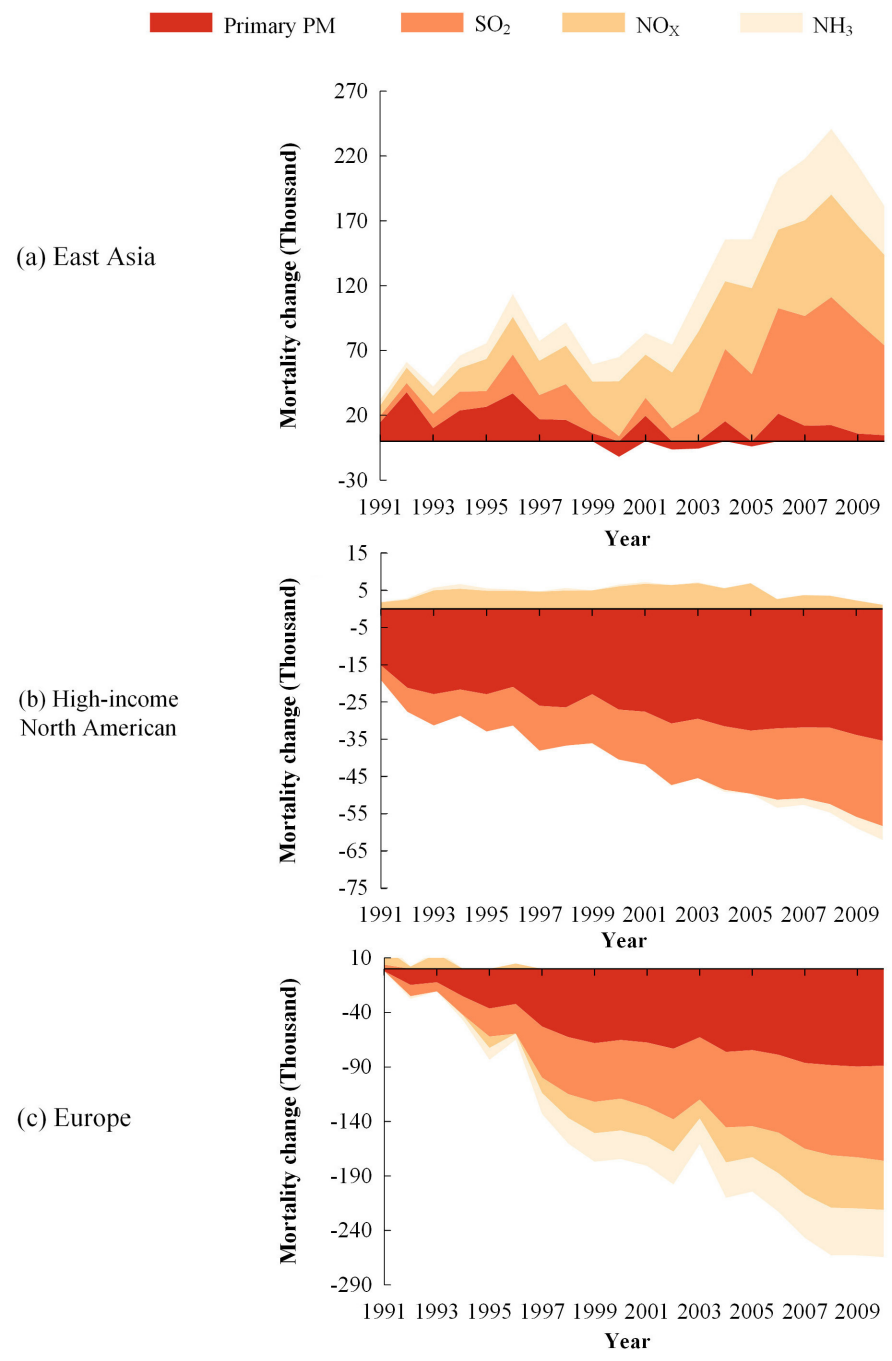
Estimated relative changes in PM_2.5_-mortality associated with changes in SO_2_, NO_x_, NH_3_,and primary PM emissions during 1991–2010 relative to the 1990 values.

Analysis of changes in PM_2.5_-mortality due to emission variations can be used to estimate the relative contributions of SO_2_, NO_x_, NH_3_, and primary PM emissions to mortalities. Figure 5 displays the relative change in PM_2.5_-mortality associated with the emission variation of these four pollutants during 1991–2010 relative to 1990. The relative contribution of emissions to PM_2.5_-mortality varied substantially in the three regions where anthropogenic emissions are the dominant sources for PM_2.5_. In East Asia, about 89% of the increase in PM_2.5_-mortality over the past two decades was associated with the increase of SO_2_, NO_x_, and NH_3_ emissions, which have not been as well controlled as primary PM emission, suggesting that the health risk arising in East Asia is mostly due to the increase in secondary inorganic aerosols. The importance of secondary inorganic sources in contributing to PM_2.5_-mortality in East Asia was also noted by Lee et al. (2015). In high-income North America, the reduction in PM_2.5_-mortality was mainly from the primary PM and SO_2_ controls, which reduced PM_2.5_-mortality by 20,000 and 19,000, respectively, from 1990 to 2010. However, such health benefits from primary PM and SO_2_ controls are partially (about 11% of the reduction in PM_2.5_-mortality was from primary PM and SO_2_ controls during 1990–2010) counteracted by the increased health risk from the growth of ammonia emissions that resulted in an increase of aerosol nitrate and ammonium concentrations. Since the sensitivity shown in Figure 5 is only a first-order approximation, the small negative NH_3_ sensitivity noted in the late 2000s is associated with the substantial reduction of NO_x_ emissions that led to the reduction of particulate ammonium. In Europe, simultaneous controls of all pollutants maximized the health benefit from the emission reductions. The PM_2.5_-mortality was reduced by 87,000, 91,000, 60,000 and 51,000 from 1990 to 2010 due to the emission mitigation of primary PM, SO_2_, NO_x_ and NH_3_, respectively.

**Figure 6:**
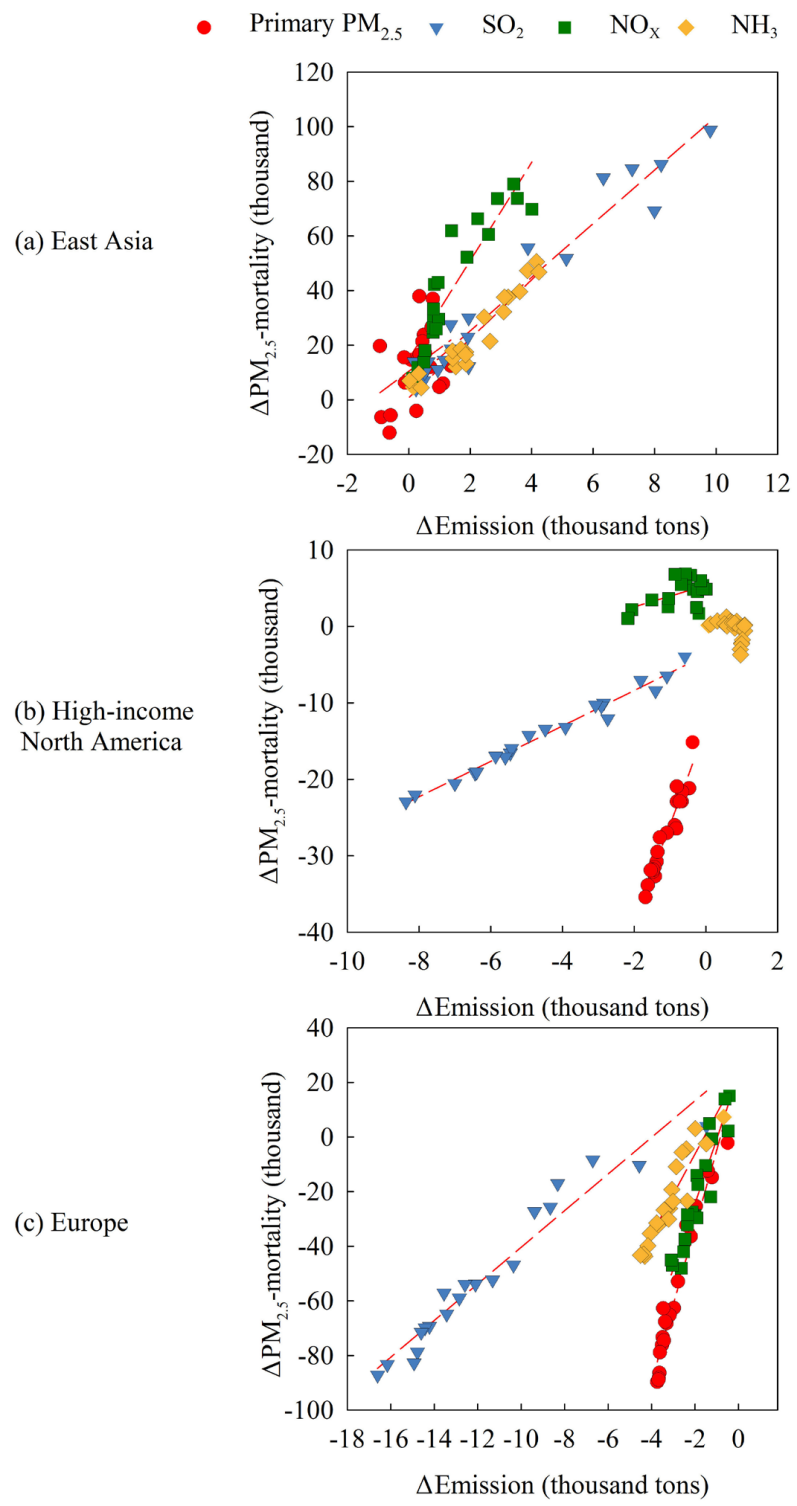
Relationship between changes in PM_2.5_-mortality and changes in emissions in three regions: (*a*) East Asia, (*b*) high-income North America, and (*c*) Europe. Each data point represents the change for each year during 1991–2010 relative to 1990. The slope of the linear regression between the variables represents the emission mitigation efficiency [i.e., emission mitigation efficiency (EME)].

The EME is a useful indicator to quantify the emission mitigation efficiency in reducing the health risk. Figure 6 presents the relationship between the change in PM_2.5_ mortalities (ΔPM_2.5_-mortality) and emissions (ΔEmission) for four pollutants in three regions during 1991–2010 relative to 1990. The EME is estimated from the linear regression of ΔPM_2.5_-mortality/ΔEmission (i.e., the slope of the scatter plots in Figure 6). In East Asia, highly linear correlations between ΔPM_2.5_-mortality and ΔEmission are noted for SO_2_ and NH_3_, and the EMEs are 9.8 and 10.8 per k-ton emissions of SO_2_ and NH_3_, respectively. NO_x_ has the highest EME (i.e., 17.9 per k-ton) among all pollutants. This is because the simultaneous increase of NH_3_ emissions facilitates the formation of aerosol nitrate thus enlarging the PM_2.5_-mortality sensitivity to NO_x_ emissions. Compared to other pollutants, the relationship between primary PM emission controls and PM_2.5_-mortality response during 1991–2010 displays no clear trend, indicating that the variation of primary PM emissions does not dominate this relationship in East Asia. As illustrated in Figure 6a, the changes in the magnitude of primary PM emissions in East Asia is much smaller than those of NO_x_, SO_2_, and NH_3_. Further, inter-annual variability in the meteorology may contribute to the variability in the estimated contribution to PM_2.5_ (and its associated mortality). Additionally, uncertainties in estimates of primary PM emissions from household combustion in East Asia could also contribute to the noted weaker relationship. However, a clear positive correlation between the PM_2.5_-mortality response and primary PM emissions was found in high-income North America, where primary PM has the highest EME (12.7 per k-ton), followed by SO_2_, with an EME of 2.3 per k-ton. Due to the increase of NH_3_ emissions over the past two decades, NO_x_ emission reductions show small or even negative PM_2.5_-related health benefits. This is because when HNO_3_ concentrations decline in response to NO_x_ emissions, the growth in NH_3_ facilitates higher partitioning of nitrate to the aerosol phase in NH_3_-limited conditions. These results are consistent with those of Fann et al. (2012) who reported that directly emitted PM_2.5_ had the highest economic value (estimated from mortalities by applying an estimate of the value of statistical life) for a 1 ton emission reduction, while NO_x_ had the lowest value, and the value for SO_2_ was in between these two. In Europe, all pollutants showed highly linear correlations with the PM_2.5_-mortality responses. The EMEs for primary PM, SO_2_, NO_x_, and NH_3_ were estimated as 28.3, 6.7, 23.7, and 14.8 per k-ton, respectively. The mitigation of primary PM was most effective for the health benefits, followed by the reduction of NO_x_ emissions which became more effective when the NH_3_ emissions were simultaneously controlled. In contrast, in high-income North America where NO_x_ emissions have declined, but NH_3_ emissions have increased, the PM_2.5_-mortality did not show a decline relative to NO_x_ emissions.

## Discussion and Conclusion

Historical trends in long-term exposure to PM_2.5_ and its impacts on premature mortality were investigated for the period of 1990–2010 across the northern hemisphere. The investigation conducted in this study was based on continuous time series of spatially resolved concentration and population data sets. Contrasting trends in PM_2.5_-mortality over the past two decades are found in developed versus developing regions. Decreasing trends in PM_2.5_-mortality are noted in Europe and high-income North America, and these reductions are mainly associated with the decrease in PM_2.5_ concentrations as well as the improvement in living conditions and the quality of medical care. In contrast, in East and South Asia, the increased health risk from the deterioration of air quality in combination with increasing population offsets the benefits from the improvement in living conditions and the quality of medical care, resulting in an increasing trend in PM_2.5_-mortality over the past two decades.

Strong positive correlations between the spatial distributions of air pollution and population are found in regions where anthropogenic emission sources are dominant. More serious health impacts from air pollution can thus be expected in regions of East Asia where both population and emissions have shown increasing trends (as in the past two decades). On the other hand, additional health benefits from air pollution controls occur when the positive correlation weakens, as witnessed in Europe and high-income North America over past two decades. To maximize the health benefits, one way to weaken such positive correlation could be by encouraging low-density residential developments, or by reducing pollution in high population regions. Such approaches could to be considered in future urban development strategies.

Mitigation of primary PM emissions appears to be the most efficient way for reducing health impacts of exposure to ambient PM_2.5_. Primary PM from combustion of household fuels could be an important contributor to PM_2.5_-mortality in East Asia. The response of PM_2.5_ concentration to NO_x_ emissions varies significantly under different NH_3_ levels (i.e., NH_3_-rich or -poor conditions) (e.g., Wang et al. 2011). Thus, the assessment of NO_x_ emission mitigation efficiency in reducing the health risk largely depends on associated measures for NH_3_ emissions. NO_x_ becomes more efficient in reducing PM_2.5_-related health risk with simultaneous control of NH_3_, as illustrated by trends in Europe. However, in conditions of declining NO_x_ but increasing NH_3_ emissions, such as in high-income North America, negligible changes in associated PM_2.5_-mortalities to these species is noted. Thus, simultaneous control of NH_3_ is necessary to maximize the health benefit from NO_x_ emission mitigation.

An assessment of VOC emission controls was not considered in this study due to the likelihood that secondary organic aerosols (SOA) were underestimated in the underlying WRF-CMAQ simulations and also because anthropogenic/biogenic splits are highly uncertain. For example, previous studies suggest that the model may underestimate the SOA formation by a factor of 2–6 (Carlton et al. 2010; Foley et al. 2010; Baek et al. 2011). Nevertheless, the importance of VOC mitigation for health benefit should be explored in the future as improvements in the model’s ability to simulate SOA are implemented.

## References

[r1] Ahmad OB, Boschi-Pinto C, Lopez AD, Murray CJ, Lozano R, Inoue M (2001). Age Standardization of Rates: A New WHO Standard. GPE discussion paper series: no.31.Geneva:World Health Organization.. http://www.who.int/healthinfo/paper31.pdf.

[r2] Appel KW, Bhave PV, Gilliland AB, Sarwar G, Roselle SJ (2008). Evaluation of the community multiscale air quality (CMAQ) model version 4.5: sensitivities impacting model performance; part II—particulate matter.. Atmos Environ.

[r3] Apte JS, Marshall JD, Cohen AJ, Brauer M (2015). Addressing global mortality from ambient PM_2.5_.. Environ Sci Technol.

[r4] Baek J Hu Y Odman MT Russell AG 2011 Modeling secondary organic aerosol in CMAQ using multigenerational oxidation of semi-volatile organic compounds. J Geophys Res 116 D22204, doi:10.1029/2011JD015911

[r5] Burnett RT Pope CA III Ezzati M Olives C Lim SS Mehta S 2014 An integrated risk function for estimating the global burden of disease attributable to ambient fine particulate matter exposure. Environ Health Perspect 122 397 403, doi:10.1289/ehp.1307049 24518036 PMC3984213

[r6] Carlton AG, Bhave PV, Napelenok SL, Edney EO, Sarwar G, Pinder RW (2010). Model representation of secondary organic aerosol in CMAQv4.7.. Environ Sci Technol.

[r7] Chen H, Goldberg MS, Villeneuve PJ (2008). A systematic review of the relation between long-term exposure to ambient air pollution and chronic diseases.. Rev Environ Health.

[r8] Chen R, Li Y, Ma Y, Pan G, Zeng G, Xu X (2011). Coarse particles and mortality in three Chinese cities: the China Air Pollution and Health Effects Study (CAPES).. Sci Total Environ.

[r9] CIESIN, CIAT (Center for International Earth Science Information Network-Columbia University; Centro Internacional de Agricultura Tropical) (2005). Gridded Population of the World, Version 3 (GPWv3): Population Density Grid.. http://dx.doi.org/10.7927/H4XK8CG2.

[r10] Dockery DW, Pope CA, Xu X, Spengler JD, Ware JH, Fay ME (1993). An association between air pollution and mortality in six U.S. Cities.. N Engl J Med.

[r11] EC-JRC (European Commission, Joint Research Centre) (2011). Emission Database for Global Atmospheric Research (EDGAR).. http://edgar.jrc.ec.europa.eu.

[r12] Fann N, Baker KR, Fulcher CM (2012). Characterizing the PM_2.5_-related health benefits of emission reductions for 17 industrial, area and mobile emission sectors across the U.S.. Environ Int.

[r13] Fann N, Fulcher CM, Hubbell BJ (2009). The influence of location, source, and emission type in estimates of the human health benefits of reducing a ton of air pollution.. Air Qual Atmos Health.

[r14] Foley KM Roselle SJ Appel KW Bhave PV Pleim JE Otte TL 2010 Incremental testing of the Community Multiscale Air Quality (CMAQ) modeling system version 4.7. Geoscientific Model Development 3 205 226, doi:10.5194/gmd-3-205-2010 PMC6104654 30147852

[r15] Henze DK Hakami A Seinfeld JH 2007 Development of the adjoint of GEOS-Chem. Atmos Chem Phys 7 2413−2433

[r16] IHME (Institute for Health Metrics and Evaluation) (2015). GBD Visualization Tool.. http://vizhub.healthdata.org/gbd-cause-patterns/.

[r17] Katsouyanni K, Touloumi G, Samoli E, Gryparis A, Le Tertre A, Monopolis Y (2001). Confounding and effect modification in the short-term effects of ambient particles on total mortality: results from 29 European cities within the APHEA2 project.. Epidemiology.

[r18] Lee CJ, Martin RV, Henze DK, Brauer M, Cohen A, van Donkelaar A (2015). Response of global particulate-matter-related mortality to changes in local precursor emissions.. Environ Sci Technol.

[r19] Lelieveld J, Evans JS, Fnais M, Giannadaki D, Pozzer A (2015). The contribution of outdoor air pollution sources to premature mortality on a global scale.. Nature.

[r20] Lim SS, Vos T, Flaxman AD, Danaei G, Shibuya K, Adair-Rohani H (2012). A comparative risk assessment of burden of disease and injury attributable to 67 risk factors and risk factor clusters in 21 regions, 1990–2010: a systematic analysis for the Global Burden of Disease Study 2010.. Lancet.

[r21] Mathur R, Gilliam R, Bullock OR Jr, Roselle S, Pleim J, Wong D, et al (2012). Extending the applicability of the Community Multiscale Air Quality model to hemispheric scales: motivation, challenges, and progress. In: *Air Pollution Modeling and its Application XXI*. NATO Science for Peace and Security Series C: Environmental Security. Steyn DG, Trini Castelli S, eds..

[r22] Mathur R, Roselle S, Young J, Kang D (2014). Representing the effects of long-range transport and lateral boundary conditions in regional air pollution models. In: *Air Pollution Modeling and its Application XXII*. NATO Science for Peace and Security Series C: Environmental Security. Steyn DG, Builtjes PJH, Timmermans RMA, eds..

[r23] Naghavi M, Wang H, Lozano R, Davis A, Liang X, Zhou M (2015). Global, regional, and national age–sex specific all-cause and cause-specific mortality for 240 causes of death, 1990–2013: a systematic analysis for the Global Burden of Disease Study 2013.. Lancet.

[r24] Pope CA, Burnett RT, Krewski D, Jerrett M, Shi Y, Calle EE (2009). Cardiovascular mortality and exposure to airborne fine particulate matter and cigarette smoke: shape of the exposure–response relationship.. Circulation.

[r25] Pope CA III Burnett RT Turner MC Cohen A Krewski D Jerrett M 2011 Lung cancer and cardiovascular disease mortality associated with ambient air pollution and cigarette smoke: shape of the exposure–response relationships. Environ Health Perspect 119 1616 1621, doi:10.1289/ehp.1103639 21768054 PMC3226505

[r26] Pope CA, Dockery DW (2006). Health effects of fine particulate air pollution: lines that connect.. J Air Waste Manage Assoc.

[r27] Pope CA, Dockery DW, Schwartz J (1995). Review of epidemiological evidence of health effects of particulate air pollution.. Inhal Toxicol.

[r28] van Donkelaar A Martin RV Brauer M Boys BL 2015 Use of satellite observations for long-term exposure assessment of global concentrations of fine particulate matter. Environ Health Perspect 123 135 143, doi:10.1289/ehp.1408646 25343779 PMC4314252

[r29] Wang J Wang S Jiang J Ding A Zheng M Zhao B 2014 Impact of aerosol–meteorology interactions on fine particle pollution during China’s severe haze episode in January 2013. Environ Res Lett 9 9 094002, doi:10.1088/1748-9326/9/9/094002

[r30] Wang J, Wang S, Voorhees AS, Zhao B, Jang C, Jiang J (2015). Assessment of short-term PM_2.5_-related mortality due to different emission sources in the Yangtze River Delta, China.. Atmos Environ.

[r31] Wang S, Hao J (2012). Air quality management in China: issues, challenges, and options.. J Environ Sci (China).

[r32] Wang S, Xing J, Jang C, Zhu Y, Fu JS, Hao J (2011). Impact assessment of ammonia emissions on inorganic aerosols in East China using response surface modeling technique.. Environ Sci Technol.

[r33] WHO (World Health Organization) (2016). Ambient (outdoor) air quality and health. Fact Sheet.. http://www.who.int/mediacentre/factsheets/fs313/en/.

[r34] Wong DC Pleim J Mathur R Binkowski F Otte T Gilliam R 2012 WRF-CMAQ two-way coupled system with aerosol feedback: software development and preliminary results. Geoscientific Model Development 5 299 312, doi:10.5194/gmd-5-299-2012

[r35] Xing J, Mathur R, Pleim J, Hogrefe C, Gan CM, Wong DC (2015a). Can a coupled meteorology-chemistry model reproduce the historical trend in aerosol direct radiative effects over the Northern Hemisphere?. Atmos Chem Phys.

[r36] Xing J, Mathur R, Pleim J, Hogrefe C, Gan CM, Wong DC (2015b). Observations and modeling of air quality trends over 1990–2010 across the Northern Hemisphere: China, the United States and Europe.. Atmos Chem Phys.

[r37] Xing J, Pleim J, Mathur R, Pouliot G, Hogrefe C, Gan CM (2013). Historical gaseous and primary aerosol emissions in the United States from 1990 to 2010.. Atmos Chem Phys.

[r38] Xing J, Zhang Y, Wang S, Liu X, Cheng S, Zhang Q (2011). Modeling study on the air quality impacts from emission reductions and atypical meteorological conditions during the 2008 Beijing Olympics.. Atmos Environ.

